# Factors Influencing Mental Health of Medical Workers During the COVID-19 Outbreak

**DOI:** 10.3389/fpubh.2020.00491

**Published:** 2020-09-22

**Authors:** Yan Zhang, Simiao Xie, Pu Wang, Guixiang Wang, Li Zhang, Xiaochen Cao, Wenzhi Wu, Yueran Bian, Fei Huang, Na Luo, Mingyan Luo, Qiang Xiao

**Affiliations:** ^1^School of Educational Science, Central China Think Tank, Huazhong University of Science and Technology, Wuhan, China; ^2^Department of Rehabilitation Medicine in the Seventh Affiliated Hospital (Shenzhen), Sun Yat-sen University, Shenzhen, China; ^3^Guangdong Engineering and Technology Research Center for Rehabilitation Medicine and Translation, Guangzhou, China; ^4^Institute of Medical Robots of Shanghai Jiao Tong University, Shanghai, China; ^5^Department of Ultrasound, Tongji Medical College, Union Hospital, Huazhong University of Science and Technology, Wuhan, China; ^6^School Hospital, Huazhong University of Science and Technology, Wuhan, China

**Keywords:** COVID-19, epidemic, mental health, medical worker, factors

## Abstract

**Background:** Since the outbreak of COVID-19, physical and psychological harm has been spreading across the global population alongside the spread of the virus. Currently, the novel coronavirus has spread to most countries in the world, and its impact on the public is also increasing. As a high-risk group in direct contact with the virus, medical workers should be monitored, and their mental health deserves extensive attention. The aim of this study was to explore the mental health of medical workers facing the novel coronavirus and the main factors affecting it.

**Methods**: The present cross-sectional study including 2,100 eligible individuals from 1,050 hospitals in China was conducted through the network platform powered by www.wjx.cn, a platform providing functions equivalent to Amazon Mechanical Turk. We used a self-designed questionnaire to collect demographic information and data on mental states, including gender, age (years), educational level, job rank, body and mind reaction, cognition of risk, and the judgment of the epidemic situation. Independent samples *t*-tests and one-way (ANOVA) analysis were carried out to compare the differences in the mental reactions according to the demographic and psychological states of the participants.

**Results**: There were 502 males (23.9%) and 1,598 females (76.1%). The participants reported feeling calm (39.1%), tense (63.0%), scared (31.4%), angry (18.8%), sad (49.0%), afraid (34.7%), optimistic (5.1%), impressed (65.0%), and confident (31.1%) during the epidemic. At the same time, the psychological stress responses of medical staff were significantly different according to the levels of exposure in their environments, duration and personal experience.

**Conclusions**: Prolonged exposure to the virus and intense work are detrimental to the mental health of medical care personnel. It is necessary to adjust work conditions and intensity according to workers' mental state flexibly and systematically.

## Introduction

At the end of 2019, a large outbreak of disease that was widespread with a high speed and a large number of infected people broke out in Wuhan (1, 2), Hubei Province, China. It spread quickly over a short period of time (3, 4), and it has been a serious threat not only to physical health (5) but also to mental health issues throughout the population (6). Since April, there have been no additional diagnoses for many days outside of Hubei, China (7), and the number of additional infections in Hubei has been largely in the double-digits, as if the Chinese epidemic were about to end. However, since the outbreak of the global epidemic (8–10), the number of imported cases has been increasing continuously, making the slightly calmer mood tense once again. If the control of imported potential patients is not adequately strong to prevent the epidemic from spreading again, previous efforts could be in vain. According to the latest real-time statistics of Johns Hopkins University, as of 08:33 Beijing time on March 16th, the cumulative number of confirmed cases of coronary pneumonia worldwide was more than 160,000, and the cumulative number of confirmed cases outside China exceeded 86,435. Studying the novel coronavirus is not only a matter of fighting COVID-19 in China but also an international public health crisis that needs to be fought by the whole world.

Since the outbreak of the epidemic, tension, anxiety and other negative emotions have spread throughout China on a large scale, so much so that people have fallen into a series of psychological crises (6). Medical care personnel, as the backbone of the front line of epidemic prevention and control, have been taking on heavy work tasks with a high risk of infection and great work pressure (11). Health-care workers, especially those in hospitals who take care of confirmed or suspected patients, are more vulnerable than the general population, experience a high risk of infection and negative emotional stress, and further risk spreading the virus to their family, friends or colleagues (6). Moreover, dangerous and susceptible environments as well as traumatic experiences caused by high exposure can all have a certain impact on an individual's emotional state and induce emotional stress responses (12) as well as severe anxiety and depressive disorders and posttraumatic stress disorder (PTSD, posttraumatic stress disorder) (11, 13). A psychological survey published in *The Lancet*· *Psychiatry* showed that the prevalence of depression, anxiety, insomnia and stress among medical staff involved in the prevention and control of the epidemic were as high as 50.7, 44.7, 36.1, and 73.4%, respectively (14).

Until now, despite the rudimentary principal notice issued by the China National Health Commission in January regarding the emergency psychological crisis intervention measures for COVID-19 pneumonia, no one has been able to provide timely and effective psychological intervention measures for medical staff.

Therefore, it is urgent and important for psychological researchers to focus on the mental health problems of medical workers during the epidemic, explore the main factors affecting their psychological stability and health, and try to prevent long-term irreversible psychological trauma to medical workers. Some scholars (15, 16) in environmental psychology have studied the effects of the environment on the individual, especially in the face of danger. According to ecological theory, the individual behavior and environment are part of an interactive ecosystem, and individual behavior has a temporal and spatial background; that is to say, there is an integrated behavioral situation (17). For the same environmental phenomenon, arousal theory argues that the influence of the same spatial and temporal background on individuals is determined by the degree of arousal experienced by any particular individual (18). The level of arousal experienced by individuals is closely related to personal experience. Inspired by this theory, this study attempted to investigate whether differences in the exposure environment, personal experience, and exposure duration of medical care personnel would lead to differences in their psychological responses, and advice and assistance were provided to personnel to prevent the development of mental health issues.

## Materials and Methods

### Participants

The questionnaire was designed for medical workers from all provinces in China. In the formal test, 2,100 medical workers were selected from 1,050 hospitals in 31 provinces to fill out the questionnaire, including 659 in Wuhan and 1,441 outside of Wuhan; 502 males and 1,598 females were included. Among them, 2.3% were under 25 years of age, 19.5% were aged 25–30, 39.5% were aged 31–40, 29.0% were aged 41–50 and 9.7% were over 50 years of age.

### Procedures

The study was designed in accordance with the tenets of the Declaration of Helsinki. Approval from the ethical authority of the School of Educational Science, Huazhong University of Science and Technology, was granted. Confidentiality and the statement confirming informed consent were managed by anonymous coding of the self-report questionnaires.

This survey used WeChat, online questionnaires and other online surveys to investigate the emotional and psychological stress states of medical staff. We used a self-designed questionnaire to collect demographics and mental state data including factors such as gender, age (years), educational level, job rank, body and mind reaction, cognition of risk, and the judgment of the epidemic situation, which was started in the third week after the outbreak, and the specific time is from February 12 to February 21, 2020. Our team sent out questionnaires through the Internet platform powered by www.wjx.cn, a platform providing functions equivalent to Amazon Mechanical Turk. Participants filled in the questionnaire on the web page through mobile phone or computer.

### Development of Psychological Stress Questionnaire

First, information was collected through small-scale interviews; next, we compiled a stress response questionnaire and determined the questionnaire topics and factors through exploratory factor analysis (EFA). Data from 312 subjects were collected as preliminary test through a web questionnaire with 15 items, including 79 in Wuhan and 233 outside Wuhan, 80 males and 232 females. Before the exploratory factor analysis, the results showed that the KMO (Kaiser–Meyer–Olkin) Measure of Sampling Adequacy was 0.765 (Chi-Square = 801.389, df = 91, *p* < 0.001), and the Bartlett's Test of Sphericity indicated that the correlation matrices on which the PCA was based were suitable for analysis. According to the factor load matrix after the rotation axis, the analysis process of the items was as follows. First, delete three items with insufficient load and which are difficult to name on each factor; next, compare the load of each item on each factor, and delete three items with small load and similar load on different factors; third, analyze each factor, and delete the items with poor division and which are difficult to explain. As per the above principles, all nine items were retained and three factors were confirmed as the result, and the total variance was 55.90%. The factors, which were named in turn, were cognition of danger (CD), reflecting the evaluation of the environmental risk of the subjects; judgment of the situation (JS), reflecting the confidence in successfully combating the epidemic and the psychology of the anti-epidemic work; and the stress reaction (SR), reflecting the physical and mental stress response produced by the subjects' current environment. See Table 1.

**Table 1:** Item loadings, eigenvalues and variances of the questionnaire according to PCA.

**Factor**	**Item**	***N***	**Loading**	**Eigenvalue**	**% of variance**
Cognition of danger (CD)	Risk of infection	Q1	0.73	2.17	24.10
	Worried about getting infected	Q6	0.66		
	The possibility of infection	Q3	0.63		
	Worried about family	Q2	0.60		
	Cognition of the current epidemic severity	Q8	0.60		
Judgment of the situation (JS)	Confidence in anti-epidemic efforts	Q9	0.84	1.38	15.38
	Fear of epidemic prevention	Q4	0.67		
Stress reaction (SR)	Sleep quality	Q5	0.74	1.28	14.21
	Need for psychological counsel	Q7	0.57		

**Figure 1:**
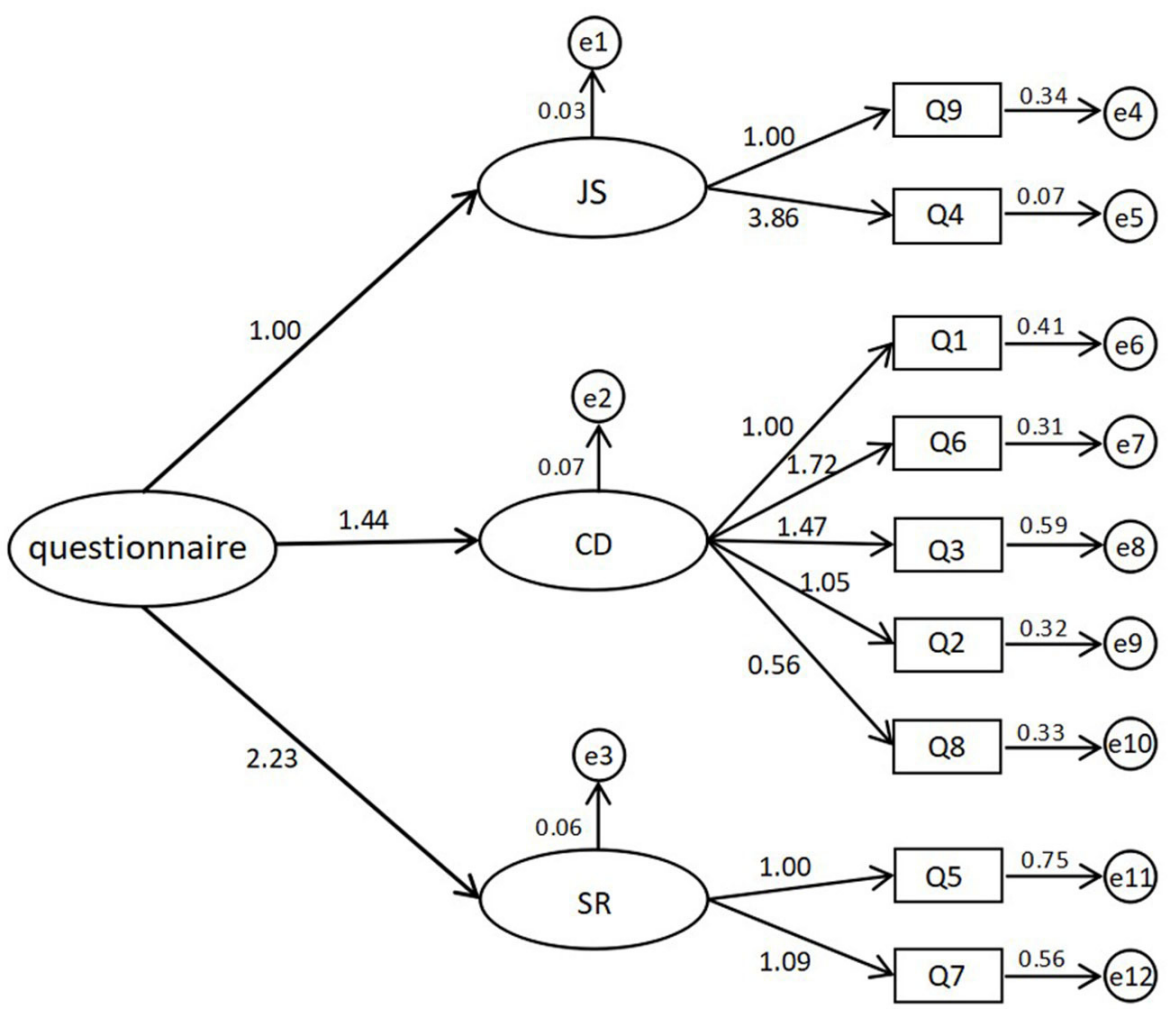
The confirmatory factors analysis (CFA) of the questionnaire.

After constructing a stress reaction questionnaire with good reliability and validity, we used confirmatory factors analysis (CFA) to confirm the validity of the questionnaire to provide a questionnaire that reflected the ideal standard. Data from 432 subjects were collected as CFA, including 118 in Wuhan and 314 outside Wuhan, 118 males and 314 females, and the fitting index tables and model diagrams drawn through Amos software of CFA are shown in Table 2 and Figure 1; finally, we conducted a wide range of formal tests.

**Table 2:** Fitting index for confirmatory factor analysis.

**Index**	**χ^**2**^**	**df**	**χ^**2**^/df**	**RMSEA**	**GFI**	**NFI**	**IFI**	**TLI**	**CFI**
Value	70.426^***^	24	2.934	0.067	0.967	0.873	0.913	0.866	0.911

*** *P < 0.001*.

The Internal Consistency Reliability (Cronbach α coefficient), partial reliability and the correlation between each factor score and the total score of the questionnaire were calculated by SPSS 23.0, and the results showed that the overall internal consistency and reliability and the overall parity factor for both was 0.67. See Table 3.

**Table 3:** Correlation analysis of each dimension of the questionnaire.

**Factor**	**CD**	**JS**	**SR**
CD	1.00	0.28^**^	0.27^**^
JS	0.27^**^	1.00	0.30^**^
*SR*	0.28^**^	0.30^**^	1.00

** *P < 0.01*.

### Data Analysis

All data analysis was carried out using SPSS 23.0 (SPSS Inc, Chicago, Illinois), and a two-tailed probability value of <0.05 was considered to be statistically significant. Descriptive statistics for the demographic and psychological states of the medical staff were shown as the mean, standard deviation (SD), number (n), and percentage. Independent samples *t*-tests and one-way (ANOVA) analysis were carried out to compare the differences in the mental reactions according to the demographic and psychological states of the participants.

## Results

### Demographic and Emotional Status

Among the 2,100 subjects who filled in the questionnaire, the distribution was not uniform, and 85.3% were doctors (1,792). According to the statistical distribution of the education level, 61.2% (1,286) of the subjects had a bachelor's degree, 19.1% (402) had a master's degree, and 5.5% (116) had a doctoral degree. Among them, there were 502 males (23.9%) and 1,598 females (76.1%). The participants primarily felt calm (39.1%), tense (63.0%), scared (31.4%), angry (18.8%), sad (49.0%), afraid (34.7%), optimistic (5.1%), impressed (65.0%) and confident (31.1%) during the epidemic.

### Differences in the Exposure Environment

In this study, the differences in the health care workers' environmental exposure were demonstrated mainly by whether they participated in the COVID-19 resistance front and had direct contact with confirmed patients. There were significant differences in the level of cognition about danger, judgment of their situation and stress reaction to the exposure environment. Specific statistical results for the medical care personnel and group comparisons are displayed in Table 4. The results show that those involved in the first-line response believed they were at greater risk of exposure to infection in the workplace(*t* = 4.872, *p* < 0.001), and they had more anxiety about infection (*t* = 2.943, *p* = 0.003), thought they were more likely to get sick (*t* = 4.295, *p* < 0.001), worried more about family infection (*t* = 1.982, *p* = 0.048), had lower confidence in obtaining victory over the epidemic (*t* = 2.339, *p* = 0.019), had poor sleep quality (*t* = 2.559, *p* < 0.001) and had a higher demand for psychological counseling (*t* = 3.491, *p* < 0.001). However, there were no significant differences for the cognition of the current epidemic severity and the fear of epidemic prevention.

**Table 4:** Differences in participation in first-line rescue.

**Factor**	**Item**	**Group (whether they participated in the front line)**	***N***	**M ± SD**	***t***	***P***
CD	Risk of infection	Yes	877	4.31 ± 0.679	4.872	<0.001
		No	1,223	4.15 ± 0.716		
	Worried about getting infected	Yes	877	4.23 ± 0.769	2.943	0.003
		No	1,223	4.12 ± 0.837		
	The possibility of infection	Yes	877	3.66 ± 0.946	4.295	<0.001
		No	1,223	3.48 ± 0.964		
	Worried about family	Yes	877	4.67 ± 0.603	1.982	0.048
		No	1,223	4.62 ± 0.658		
	Cognition of the current epidemic severity	Yes	877	4.51 ± 0.626	0.936	0.349
		No	1,223	4.49 ± 0.600		
JS	Confidence in anti-epidemic efforts	Yes	877	1.52 ± 0.653	2.339	0.019
		No	1,223	1.45 ± 0.607		
	Fear of epidemic prevention	Yes	877	2.96 ± 0.903	0.188	0.851
		No	1,223	2.95 ± 0.905		
SR	Sleep quality	Yes	877	2.71 ± 1.023	3.559	<0.001
		No	1,223	2.56 ± 0.935		
	Need for psychological counsel	Yes	877	2.27 ± 0.914	3.491	<0.001
		No	1,223	2.13 ± 0.896		

### Differences in Personal Experience

The differences in health care workers' personal experiences were affected mainly by whether they had experienced SARS or another epidemic. There were significant differences in the levels of cognition of danger, judgment of their situation and stress reactions to personal experiences. Specific statistical results for the medical care personnel and group comparisons are displayed in Table 5, which shows that medical staff involved in SARS prevention believed they had a greater risk of exposure to infection in the workplace (*t* = 2.220, *P* = 0.027), were more likely to be infected (*t* = 2.057, *p* = 0.040), had more confidence in the success in epidemic prevention and control (*t* = −2.895, *p* = 0.004), less fear of fighting the epidemic (*t* = −3.167, *p* = 0.002), and poor sleep quality (*t* = 2.848, *p* = 0.004). However, there were no significant differences for the items regarding being worried about getting infected, the cognition of the current epidemic severity and the need for psychological counseling.

**Table 5:** Differences in experience with SARS or other outbreaks.

**Factor**	**Item**	**Group (whether experienced SARS or other outbreaks)**	***N***	**M ± SD**	***t***	***P***
CD	Risk of infection	Yes	1,202	4.25 ± 0.716	2.220	0.027
		No	898	4.18 ± 0.687		
	Worried about getting infected	Yes	1,02	4.17 ± 0.818	0.199	0.842
		No	898	4.16 ± 0.802		
	The possibility of infection	Yes	1,202	3.59 ± 0.974	2.057	0.040
		No	898	3.50 ± 0.940		
	Worried about family	Yes	1,202	4.66 ± 0.627	1.526	0.127
		No	898	4.62 ± 0.648		
	Cognition of the current epidemic severity	Yes	1,202	4.52 ± 0.601	1.920	0.055
		No	898	4.47 ± 0.623		
JS	Confidence in anti-epidemic measures	Yes	1,202	1.44 ± 0.604	−2.895	0.004
		No	898	1.52 ± 0.654		
	Fear of epidemic prevention	Yes	1,202	2.90 ± 0.923	−3.167	0.002
		No	898	3.03 ± 0.873		
SR	Sleep quality	Yes	1,202	2.68 ± 1.007	2.848	0.004
		No	898	2.56 ± 0.928		
	Need for psychological counsel	Yes	1,202	2.19 ± 0.926	0.086	0.932
		No	898	2.19 ± 0.879		

### Differences in Exposure Duration

Since the outbreak of the epidemic, medical workers have been stressed and made to work for long periods of time, with little time for rest. The difference in exposure duration was reflected mainly by the number of continuous working days. This study compared the differences in the duration of the participants' operational time in medical care work and divided the working hours into four levels for horizontal comparison, which found that the longer the working hours were, the more likely the participants believed they would be infected (*F* = 5.868, *P* < 0.001), the more worried they were about family members being infected (*F* = 2.870, *P* < 0.035), and the poorer their sleep-quality was (*F* = 18.403, *P* < 0.001). However, the fear of epidemic prevention was lower (*F* = 6.052, *P* < 0.001). Furthermore, there were significant fluctuations in two dimensions, cognition of the current epidemic severity (*F* = 2.676, *P* = 0.046) and confidence in anti-epidemic measures (*F* = 11.275, *P* < 0.001), caused by the increase in working hours, which at first declined a certain degree, then increased significantly. See Table 6.

**Table 6:** Differences in the length of work.

**Factor**	**Item**	**Group (work time)**	***N***	**M ± SD**	***F***	***P***
CD	Risk of infection	Within 3 days	222	4.18 ± 0.811	1.910	0.126
		4–7 days	502	4.17 ± 0.670		
		8–14 days	709	4.22 ± 0.684		
		More than 15 days	667	4.26 ± 0.712		
		total	2,100	4.22 ± 0.704		
	Worried about getting infected	Within 3 days	222	4.14 ± 0.839	1.225	0.299
		4-7 days	502	4.23 ± 0.686		
		8–14 days	709	4.16 ± 0.814		
		More than 15 days	667	4.14 ± 0.881		
		total	2,100	4.17 ± 0.811		
	The possibility of infection	Within 3 days	222	3.37 ± 1.037	5.868	<0.001
		4–7 days	502	3.52 ± 0.0.900		
		8–14 days	709	3.53 ± 0.0.935		
		More than 15 days	667	3.66 ± 0.994		
		total	2,100	3.55 ± 0.960		
	Worried about family	Within 3 days	222	4.59 ± 0.692	2.870	0.035
		4–7 days	502	4.65 ± 0.619		
		8–14 days	709	4.60 ± 0.674		
		More than 15 days	667	4.69 ± 0.583		
		total	2,100	4.64 ± 0.636		
	Cognition of the current epidemic severity	Within 3 days	222	4.57 ± 0.548	2.676	0.046
		4–7 days	502	4.51 ± 0.595		
		8–14 days	709	4.45 ± 0.646		
		More than 15 days	667	4.52 ± 0.601		
		total	2,100	4.50 ± 0.611		
JS	Confidence in anti-epidemic measures	Within 3 days	222	1.48 ± 0.622	11.275	<0.001
		4–7 days	502	1.58 ± 0.684		
		8–14 days	709	1.50 ± 0.624		
		More than 15 days	667	1.37 ± 0.572		
		total	2,100	1.48 ± 0.627		
	Fear of epidemic prevention	Within 3 days	222	3.07 ± 0.858	16.052	<0.001
		4–7 days	502	3.11 ± 0.819		
		8–14 days	709	2.99 ± 0.882		
		More than 15 days	667	2.77 ± 0.970		
		total	2,100	2.96 ± 0.904		
SR	Sleep quality	Within 3 days	222	2.28 ± 0.925	18.403	<0.001
		4–7 days	502	2.52 ± 0.947		
		8–14 days	709	2.65 ± 0.934		
		More than 15 days	667	2.80 ± 1.018		
		total	2,100	2.63 ± 0.976		
	Need for psychological counsel	Within 3 days	222	2.14 ± 0.897	0.385	0.764
		4–7 days	502	2.21 ± 0.883		
		8–14 days	709	2.19 ± 0.874		
		More than 15 days	667	2.19 ± 0.959		
		total	2,100	2.19 ± 0.906		

## Discussion

Since the emergence of the new coronavirus pneumonia in Wuhan at the end of December 2019, numerous medical staff have been working intensively for nearly 3 months and will continue to do so in the future. The results showed that the current mental health status of health care workers was not stable, with a general mean of more than 3.5 in terms of the cognition of danger, and most of the mean values were above 4 (according to Richter's five-point score, which gradually declined from 1 to 5). Regarding the dimensions of the judgment of the situation and the stress reaction, the medical staff were optimistic, and there was no obvious negative somatization phenomenon. It was found that the exposure environment, personal experience, and exposure duration had significant effects on the psychological stress and emotional responses of medical staff.

Medical workers involved in the front-line of prevention were affected to different degrees in these three dimensions, and the statistical level was significantly different. This may be due to direct exposure to close contact with the virus and negative tension in their environment as well as the fear of threats to their own lives. Additionally, the medical work environment is infested with patients' senses of grief and panic, resulting in a constant psychological burden for front-line medical workers. At the same time, there is no clear and targeted cure for the novel coronavirus infection. Doctors and nurses are not in a position to cope with the suffering of infected patients, which is further increasing their psychological burden.

The influence of medical workers who have experienced SARS and other epidemic diseases was not synchronized in these three dimensions. In the dimension of the cognition of danger, employees with experience of SARS and other epidemic prevention situations felt more serious psychological pressure, while for the dimension of the judgment of the situation, they had more confidence about overcoming this epidemic. This may be explained by the success of the prevention and control of infectious diseases like SARS, which has enhanced the collective sense of the efficacy of health care groups in the face of similar diseases, thus enhancing their confidence. However, the difficulties of living through that process and the negative emotions experienced are difficult to describe, and the impact has not gradually disappeared over time. The outbreak of the epidemic quickly awakened the former unhappy memory, so the iteration and development of risk cognition were derived from a certain preexisting foundation. This is also a wake-up call for psychological workers to remind us to do a good job of psychological intervention and health care even after illness.

As the time of exposure to the virus increases, the mental state of the medical staff deteriorates. Regarding the factor of risk cognition, the negative psychological state of the medical staff gradually intensifies with the passage of time, whereas the optimistic hope dimension presents the inverted U curve change. In the physical and mental response dimension, the sleep-quality of the medical staff is generally poor, but the difference in the level of demand for psychological counseling is not significant. This may be because, in the early days of the outbreak, a large number of patients poured into hospital emergency rooms and fever outpatient departments, increasing the already heavy workload and responsibility of all medical staff. Meanwhile, the high intensity of work continued without rest, there were inadequate protective supplies and protective isolation measures, the outpatient procedure organization became cluttered, and other phenomena have continually aggravated the psychological burden of medical staff, reducing the confidence of medical workers in prevention and control. As the epidemic situation gradually comes under control, medical work tends to stabilize, so the confidence in prevention and control has been steadily recovered. However, the negative feelings of health care workers have not been effectively vented, such as the grievances, fears, and powerlessness of medical staff in the face of dissatisfaction from patients and their families because of the lack of timely treatment. The inner suffering cannot slowly dissipate over time. By contrast, it is highly likely that the backlog of negative emotions causes some mental health issues, especially PTSD, requiring the attention of psychological workers.

### Suggestion and Contribution

PTSD usually occurs within a few weeks of traumatic events but can also appear after a few months or even a few years, and the duration is usually half a year or more (19–21), depending on the severity of the event and the individual state of mind (22, 23). The current trend of the epidemic situation in China has been obviously controlled, and the tension of the medical staff can be relaxed in stages, which is the best time for online psychological guidance. Moreover, the outbreak of foreign epidemics is rapid, and many countries lack the experience of prevention and control. China plans to send some supportive medical workers to countries where the epidemic is ongoing. The relief of tension is about to face new challenges, and it is essential to effectively perform psychological intervention and regulation for medical staff. Both Chinese and international mental health workers must pay attention to this problem and stabilize psychological security (24, 25).

This study found that the psychological state of medical workers was significantly affected by the high-risk environment of direct contact infection, long working hours, and personal experiences. However, the only factors that can be controlled are the working environment and working hours. The authors suggests the establishment of a matching system between the psychological state and the working intensity of medical staff; after all, only upon a foundation of psychological security can the work be completed efficiently. The psychological security work needs to be carried out in a systematic and hierarchical manner from the local level to a more general investigation by utilizing close attention to ensure that every corner of the mental health of medical staff is explored. First, based on the overall comprehensive investigation, a medical staff psychological state tracking system should be established. Second, all mental state files should be classified into attention levels, such as core, focus, general attention, etc. Meanwhile, each health worker will be assigned a psychologist who is responsible for paying regular attention to their mental health problems. Psychological workers need to evaluate whether the medical staff's work schedule matches their psychological status and periodically review their appropriate work intensity level. Finally, specific psychological interventions need to be carried out for all health workers who are marked as working at a certain level of focus and above by recording any incidents in their mental state file.

## Conclusion

By investigating the emotional and psychological stress responses of medical staff during the prevention and control of the new coronary pneumonia, it was found that the high intensity of medical work had a variety of negative effects on their risk cognition, confidence in overcoming the epidemic situation and physical and mental reactions, all of which are detrimental to the mental health of medical staff. In addition, the exposed environment, personal experiences and differences in the length of their work hours played important roles. To maintain the mental health and stability of medical staff and avoid the influence of mental health issues like PTSD, psychological workers need to take targeted measures to systematically solve the mental health problems of medical workers in the face of major infectious disease crises.

## Data Availability Statement

The raw data supporting the conclusions of this article will be made available by the authors, without undue reservation.

## Ethics Statement

Approval from the ethical authority of the School of Educational Science, Huazhong University of Science and Technology, was granted. Confidentiality and the statement confirming informed consent were managed by anonymous coding of the self-report questionnaires. The patients/participants provided their written informed consent to participate in this study.

## Author Contributions

YZ, PW, and LZ conceived and designed the questionnaire. LZ recruitment and payment of participants. SX, GW, XC, YB, FH, NL, ML, and QX analyzed the data. SX wrote and revised the paper. All the authors have approved the manuscript and agreed with submission to your esteemed journal.

## Conflict of Interest

The authors declare that the research was conducted in the absence of any commercial or financial relationships that could be construed as a potential conflict of interest.
